# Beyond distress relief: the Anhedonic Subtype of nonsuicidal self-injury and the imperative for Positive Affect Treatment

**DOI:** 10.3389/fpsyt.2026.1837385

**Published:** 2026-05-07

**Authors:** Cheng-Han Li, Jing Qian, Jing-Ze Qian, Xiao-Hong Wang, Qian-Qian Zhang

**Affiliations:** 1 Third Affiliated Hospital of Wenzhou Medical University, Wenzhou, China; 2 Wenzhou Medical University, Wenzhou, China

**Keywords:** adolescents, anhedonia, nonsuicidal self-injury (NSSI), Positive Affect Treatment (PAT), reward processing

## Abstract

This perspective article argues that the theoretical landscape of nonsuicidal self-injury (NSSI) has long been stabilized by the “hydraulic” model of Automatic Negative Reinforcement, which conceptualizes self-harm primarily as a mechanism to down-regulate aversive hyper-arousal. While this framework successfully elucidates the etiology of self-injury driven by high-intensity negative affect, it fails to account for a substantial, treatment-resistant phenotype: adolescents driven by profound anhedonia and ventral striatal hypofunction. This perspective article argues for the formal recognition of an “Anhedonic Subtype” of NSSI. Synthesizing recent epidemiological data identifying “emptiness” as a central symptom network bridge, alongside neurobiological evidence of reward blunting, we posit that for this subtype, NSSI functions not as a sedative, but as a mechanism of “forced activation.” We propose a preliminary differential diagnostic framework distinguishing defensive dissociation from anhedonic deficit and outline the theoretical rationale for exploring a shift in clinical intervention from distress tolerance toward positive affect up-regulation. The clinical utility of this framework remains to be evaluated in future empirical research.

## 1 Introduction

For nearly two decades, the scientific understanding of nonsuicidal self-injury (NSSI) has been anchored by the Emotion Regulation Paradigm. Crystallized by the foundational work of Matthew Nock and colleagues, this framework posits that self-injury is not a random act of destruction but a functional behavior maintained by specific reinforcement processes. The field has largely codified this understanding through the Four-Function Model (FFM), which serves as the standard diagnostic taxonomy (1). This model categorizes NSSI along two intersecting axes, namely the source of reinforcement (intrapersonal versus interpersonal) and the valence of the change (negative versus positive). This matrix yields four distinct drivers: Automatic Negative Reinforcement (ANR), Automatic Positive Reinforcement (APR), Social Negative Reinforcement (SNR), and Social Positive Reinforcement (SPR) (2).

More recently, Carenys and Adan (3) have proposed that NSSI may be more parsimoniously understood through the lens of behavioral addiction, emphasizing tolerance, compulsive urges, and reward system dysregulation as core maintaining mechanisms. This addiction framework provides a complementary perspective that, as we argue below, is particularly relevant to the Anhedonic Subtype.

Despite the theoretical breadth of the FFM, clinical conceptualization has become heavily skewed toward a single quadrant, namely Automatic Negative Reinforcement. This “hydraulic” model views the psyche as a pressurized system where affective intensity builds until it breaches the individual’s window of tolerance. In this high-arousal state, the act of cutting or burning functions as a somatic release valve, rapidly down-regulating physiological and subjective distress to restore homeostasis. The dominance of this model is not arbitrary but is supported by extensive ecological momentary assessment (EMA) data where “to stop bad feelings” consistently ranks as the most endorsed motive for NSSI (4–6). Consequently, the “gold standard” clinical interventions, most notably Dialectical Behavior Therapy (DBT), have been engineered to address this specific deficit (7–9). By targeting “distress tolerance,” these protocols train patients to endure high-intensity negative affect without resorting to maladaptive behaviors.

**Figure 1:**
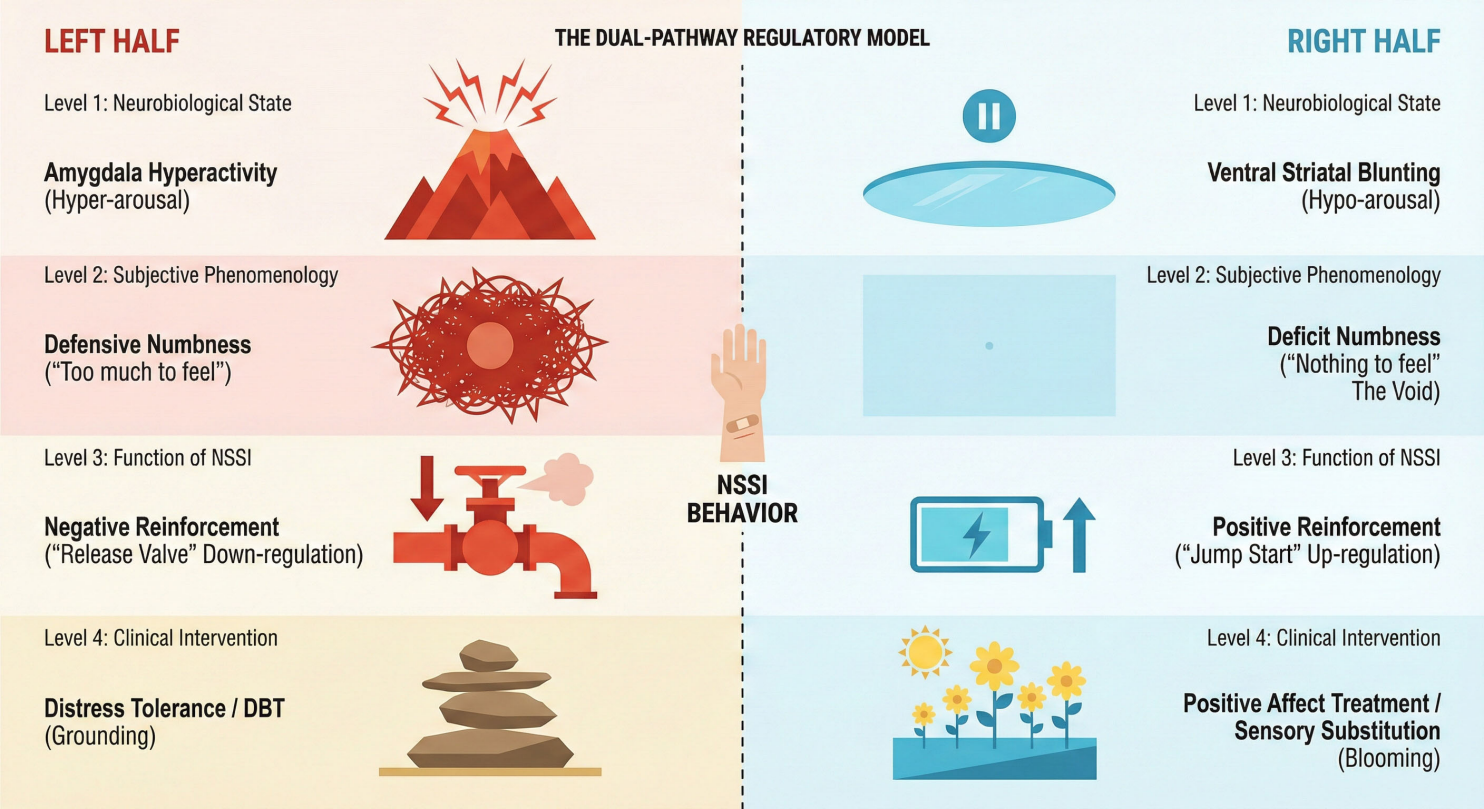
The dual-pathway regulatory model of nonsuicidal self-injury.

However, despite the undeniable efficacy of these protocols for a significant portion of the patient population, a stubborn clinical dilemma persists. Meta-analytic reviews indicate that a substantial subset of adolescents exhibits “treatment resistance,” failing to respond to standard emotion-regulation protocols (10, 11). These patients acquire distress tolerance skills yet continue to self-injure, suggesting that their behavior is not driven by an inability to tolerate distress, but by a fundamentally different driver (12). Detailed clinical phenomenology reveals that for these adolescents, the antecedent to self-injury is not a “fire” to be extinguished, but a “void” to be filled. **Figure 1** provides an overview of the proposed Dual-Pathway Regulatory Model, distinguishing distress-driven NSSI from an anhedonia-driven “forced activation” pathway and motivating the shift toward positive affect up-regulation.

The present article is a Perspective that advances a theoretical argument rather than a systematic or narrative review of the literature. Our objective is to synthesize converging evidence from epidemiology, network analysis, neuroimaging, and clinical science to propose the formal recognition of an “Anhedonic Subtype” of NSSI and to outline the preliminary theoretical basis for a corresponding shift in clinical intervention. The literature cited herein was selected to build this specific argument and is not intended to represent an exhaustive survey of the field.

## 2 Identifying the Anhedonic Subtype

### 2.1 The epidemiology of the void

Recent epidemiological surveillance and network analysis data challenge the historical view that anhedonia is merely a secondary feature of depression, revealing it instead as a primary, distinct driver of self-harm (13). While the reduction of negative affect remains the most common function of NSSI, with the strength of this “distress-function” relationship often contingent on various moderators (14), the drive to generate feeling, Automatic Positive Reinforcement (APR), is far more prevalent than clinical heuristics often suggest. Data indicate that among adolescents with NSSI, endorsement of Automatic Positive Reinforcement ranges from 29.3% to 46.9% in clinical samples, with over half of self-injurers in community samples reporting that they have engaged in the behavior to “feel something” or “generate sensation”.

The predictive power of this “emptiness” is statistically profound. A 2025 machine-learning enhanced EMA study identified subjective emptiness as one of the top three predictors of NSSI thoughts, ranking alongside anxiety and loneliness. Crucially, the study calculated that every one-unit increase in subjective emptiness corresponded to a 24% increase in the odds of experiencing NSSI thoughts (15). This effect size rivals that of high-arousal anxiety, suggesting that the absence of feeling is as potent a trigger as the excess of it. Furthermore, network analysis of adolescents with comorbid depression and NSSI has identified anhedonia not just as a peripheral symptom, but as a “bridge symptom”—a central node that connects depressive withdrawal with behavioral urgency (16, 17). These findings indicate that for a specific subtype of patients, the pathway to self-injury is paved not by sadness, but by the inability to feel pleasure.

### 2.2 The Anhedonic Subtype

In light of this evidence, we propose the formal recognition of a distinct “Anhedonic Subtype” of NSSI. For this subtype, the primary driver of self-injury is anhedonia—a transdiagnostic deficit in the reward system that renders the individual incapable of deriving pleasure or meaning from normative stimuli (18). The core hypothesis of this perspective is that for the Anhedonic Subtype, NSSI functions as a maladaptive form of “forced activation” or sensation seeking. Therefore, treating these patients with “distress tolerance” is fundamentally misaligned. Telling a patient who feels “dead inside” to tolerate their state may be ineffective when their primary complaint is a lack of vitality; they do not need to calm down, they need to wake up.

To resolve this clinical mismatch, we must dismantle the monolithic concept of “numbness.” While patients often use the word indiscriminately, neurobiological reality suggests two distinct states (19, 20). The first, Defensive Numbness, aligns with traditional trauma-informed models of dissociation. Here, numbness is a secondary reaction to primary distress—a defense mechanism triggered when emotional intensity breaches the window of tolerance (21). Physiologically, this often manifests as “detached arousal,” where the individual is subjectively detached but remains in a state of high sympathetic arousal beneath the dissociative suppression. For this group, NSSI serves an “anti-dissociation” function, piercing the barrier to restore homeostasis (22).

In stark contrast, the proposed Anhedonic Subtype exhibits Deficit Numbness. Here, numbness is the primary state, not a reaction to stress. It is a deficit, not a defense (23). Characterized by a hypo-functioning ventral striatum, the individual feels “dead inside” because they are chronically under-stimulated. Physiologically, this presents as low arousal, blunted autonomic response, and flattened affect. For this group, the function of NSSI is “forced activation.” (24, 25). They engage in self-injury to generate any feeling, even pain, because the “pain of feeling nothing” is more intolerable than the physical injury itself. The goal is not homeostasis, but stimulation.

## 3 Neurobiological mechanisms of forced activation

### 3.1 Pain-offset relief and dopaminergic recruitment

The validity of the Anhedonic Subtype rests on understanding the specific neurobiological mechanisms that allow pain to function as a reward substitute. The primary mechanism lies in the “Pain-Offset Relief” theory. Standard reward processing involves the release of dopamine in the nucleus accumbens (ventral striatum) in response to positive stimuli; however, in anhedonic individuals, this system is blunted, and normative stimuli fail to elicit a response. Conversely, the removal of pain is a potent, evolutionarily conserved reward mechanism. Research demonstrates that the cessation of a painful stimulus triggers a sharp spike in positive affect and a release of endogenous opioids and dopamine (26). For a patient with a “starved” reward system, this pain-offset induced dopamine spike may be the only accessible form of euphoria available to them.

Recent data confirms this dynamic, patients with high anhedonia report that after NSSI, they do not merely feel a reduction in negative affect; they feel a significant increase in *positive* affect, describing sensations of relief, calm, and even euphoria (27). This “relief high” reinforces the behavior via positive reinforcement. This behavioral mechanism is underpinned by structural deficits. Neuroimaging studies consistently show that adolescents with NSSI, particularly those with depressive comorbidity, exhibit significant hypoactivation in the ventral striatum during reward anticipation. When presented with a potential reward, the healthy adolescent brain activates with anticipation; the brain of the anhedonic self-injurer remains metabolically quiet (28). This neural “silence” manifests subjectively as the void, rendering traditional behavioral activation strategies ineffective and necessitating the high-intensity input of self-injury to “jump-start” the stalled reward system.

### 3.2 The Opioid Deficiency Hypothesis: addiction to the cure

While the blunted dopaminergic response explains the *lack* of anticipatory pleasure (“wanting”), it is the dysregulation of the endogenous opioid system that likely explains the *maintenance* of the behavior (“liking” and relief). This distinction leads to the “Opioid Deficiency Hypothesis” of anhedonic NSSI. Under homeostatic conditions, social connection and normative rewards stimulate the release of β-endorphins, providing a background sense of well-being (29). However, evolutionary neuroscience suggests that the neural pathways processing physical pain and social separation are anatomically shared (the dorsal anterior cingulate cortex) (30). For the anhedonic adolescent, who often suffers from profound social disconnection (“Social Anhedonia”), the system is starved of the natural opioids typically released through social bonding.

In this state of “opioid withdrawal,” NSSI functions as a high-potency chemical correction. When skin is cut or burned, the body mounts a massive analgesic response, flooding the system with endogenous opioids to mitigate the injury. For the opioid-starved brain, this sudden influx functions not merely as pain relief, but as a “chemical prosthesis” for social connection—mimicking the neurobiology of comfort and warmth (31). Recent positron emission tomography (PET) studies have demonstrated that chronic self-injurers exhibit significant dysregulation of the endogenous opioid system, characterized by altered mu-opioid receptor availability in regions such as the orbitofrontal cortex (32). While some evidence suggests a compensatory upregulation of receptor availability due to severe endogenous opioid deficits, the overall profile of reward circuit impairment remains strikingly similar to that observed in substance use disorders and other psychiatric conditions involving anhedonia (33). Consequently, the behavior is reinforced by an addiction-like cycle where chronic self-injury serves as a negative reinforcement mechanism to alleviate a baseline “opioid deficit”. This process may involve the repeated release of endorphins that leads to neuroadaptive changes in receptor sensitivity, deepening baseline anhedonia and necessitating more frequent or severe injury to achieve the same affective regulation—a phenomenon described as the “escalation of lethality”.

This addiction-like trajectory is consistent with the broader framework proposed by Carenys and Adan (3), whose systematic review marshals evidence from subjective experience, diagnostic criteria overlap, and neurobiological substrates to argue that NSSI may function as a behavioral addiction. Their model highlights tolerance development, withdrawal-like dysphoric states, and progressive behavioral escalation as hallmarks of addictive NSSI. Critically, their observation that the reinforcement function of NSSI may shift over time—from positive reinforcement (generating relief or euphoria) toward negative reinforcement (escaping withdrawal-like aversive states)—maps directly onto the trajectory we propose for the Anhedonic Subtype.

### 3.3 The biological substrate of the void

The neurobiological portrait of the Anhedonic Subtype extends beyond the initial failure to register reward; it encompasses a systemic failure to *sustain* positive affect. This phenomenon, clinically termed “Positive Affect Dampening,” represents a critical regulatory deficit. While healthy individuals engage in “savoring”, the capacity to attend to, appreciate, and elongate positive emotional experiences (34), adolescents with anhedonic NSSI exhibit a cognitive style that actively curtails positive moments. Research indicates that even when a reward signal breaches the threshold of the blunted ventral striatum, the regulatory system immediately suppresses it with dampening cognitions such as “this won’t last” or “I don’t deserve this” (35). Consequently, the window of positive affect is prematurely closed, returning the individual to the void. This explains the failure of simple behavioral interventions; these patients lack the “neural velcro” required to make positive experiences stick, rendering fleeting moments of joy insufficient to counteract the pervasive deadness.

Recent biomarker research has further grounded this “void” in physiology, specifically through the lens of immunopsychiatry. Adolescents with NSSI and depressive comorbidity exhibit elevated levels of pro-inflammatory cytokines, including C-reactive protein (CRP) and Interleukin-6 (IL-6) (36). These markers are not merely systemic indices of stress; they are directly correlated with corticostriatal dysconnectivity, effectively disrupting the communication lines between the “thinking” prefrontal cortex and the “feeling” striatum (37). This inflammatory profile induces “sickness behavior,” a conserved evolutionary response characterized by fatigue, social withdrawal, and profound anhedonia. Thus, the “emptiness” reported by these patients is not a metaphor but a somatic reality driven by neuroinflammation. The act of self-injury, therefore, may be understood as a desperate attempt to break through this inflammatory haze, using the sharp shock of pain to temporarily override the sickness response and force a sense of vitality.

## 4 Developmental and lethality risks

### 4.1 The adolescent crucible

To fully understand the emergence of the Anhedonic Subtype, we must situate it within the developmental biology of adolescence. This period is characterized by a “Dual Systems Mismatch,” where the maturation of the subcortical reward regions (e.g., ventral striatum) outpaces the top-down control of the prefrontal cortex. Typically, this imbalance manifests as risk-taking and hypersensitivity to reward. However, recent longitudinal imaging suggests a “synaptic pruning error” in depressive phenotypes. During the extensive synaptic remodeling of adolescence, an over-pruning of dopaminergic receptors or a decoupling of the frontostriatal loops can lead to a precipitous drop in basal reward sensitivity (38).

This creates a specific developmental trap: the adolescent brain is biologically programmed to seek high-intensity sensation to facilitate independence, yet the anhedonic adolescent lacks the neural hardware to process normative rewards (like social acceptance or academic success). This “Reward Expectancy Gap,” the chasm between the biological drive for sensation and the inability to experience it, creates an intolerable state of tension (39). NSSI exploits this gap. Because pain processing pathways mature earlier and are distinct from social reward pathways, self-injury becomes a reliable, “low-cost” method to hack the reward system when normative pathways are pruned or functionally silent (40, 41). Thus, the Anhedonic Subtype is not merely a psychological state but a neurodevelopmental artifact of a reward system that has failed to come online during its critical period.

Although the present Perspective focuses on adolescents, for whom the neurodevelopmental context creates a particularly acute vulnerability, the core mechanisms proposed for the Anhedonic Subtype are not inherently age-limited. Ventral striatal hypofunction, endogenous opioid dysregulation, and positive affect dampening have been documented across the adult lifespan in the context of major depression, schizophrenia, and substance use disorders (18). Adult populations with chronic NSSI—particularly those with comorbid borderline personality disorder or treatment-resistant depression—may similarly exhibit an anhedonia-driven “forced activation” pathway. Indeed, the behavioral addiction framework proposed by Carenys and Adan (3) draws primarily on adult samples, suggesting that the tolerance and escalation dynamics we describe may be equally operative beyond adolescence. We focus on adolescents here because the developmental mismatch between reward-seeking drive and reward-processing capacity (the “Reward Expectancy Gap”) is most pronounced during this period, and because adolescence represents the peak window for NSSI onset and thus the most critical period for early intervention. Future research should explicitly test whether the Anhedonic Subtype and its proposed treatment implications generalize to adult self-injurers.

### 4.2 The lethal intersection: anhedonia and the acquired capability for suicide

Recognizing the Anhedonic Subtype is critical for suicide prevention, as this phenotype appears to carry a unique lethality risk. The Interpersonal Theory of Suicide posits that lethal self-harm requires both the *desire* to die and the *capability* to enact lethal violence (42). While depressive sadness contributes to the desire, it is anhedonia and NSSI that dangerously build the “Acquired Capability.”

Adolescents in this subtype occupy a lethal intersection. Their chronic state of emotional deadness erodes the instinctual fear of death, as life ceases to feel inherently valuable or potent. Simultaneously, their habitual use of NSSI systematically habituates them to physical pain and bodily destruction. Recent meta-analytic evidence indicates that anhedonia is robustly associated with current suicidal ideation, with individuals exhibiting anhedonic symptoms showing significantly elevated suicide risk compared to those without anhedonia, even when controlling for depression severity and psychiatric diagnoses (43). Unlike anxious patients who may be protected by their own fear of pain or death, the anhedonic patient’s indifference to survival, combined with a practiced ability to override the body’s preservation instincts, creates a “silent” but highly lethal clinical profile. This suggests that clinicians should carefully evaluate the combination of “emptiness” and “cutting” not just as a cry for help, but as a rehearsal for death.

## 5 From down-regulation to up-regulation

### 5.1 The mismatch of traditional distress tolerance

The recognition of the Anhedonic Subtype necessitates a paradigm shift in clinical intervention. The current hegemony of “Distress Tolerance” models creates a fundamental mismatch for these patients. Standard safety planning and DBT protocols are predicated on the assumption of hyper-arousal; they aim to calm the patient down. However, treating a Type II (Anhedonic) patient with relaxation strategies or paced breathing is akin to treating a starving person by teaching them to tolerate hunger pangs. It manages the symptom but ignores the deficit. If the void remains, the drive for “forced activation” via NSSI will persist because the patient is not seeking safety; they are seeking sensation. Consequently, the clinical goal may need to shift from the containment of harm toward the revitalization of affect, moving from Down-Regulation protocols to Up-Regulation protocols.

### 5.2 Implementing Positive Affect Treatment

This shift requires the integration of principles from PAT, a therapeutic modality designed specifically to target reward sensitivity. Unlike traditional Behavioral Activation (BA), which focuses on increasing the frequency of mastery or pleasure activities, an Up-Regulation approach focuses on “Reward Prediction and Exposure.” In this framework, we hypothesize that patients would not simply engage in activities but would be trained to explicitly predict the level of enjoyment they expect, engage in the activity, and then compare the actual experience to their prediction. This process leverages “prediction error learning” to force the recalibration of the ventral striatum (44). By repeatedly highlighting the discrepancy between the expected “void” and the actual experience of even a minor reward, the brain is retrained to notice and value positive stimuli, gradually shifting the neural baseline away from anhedonia.

While PAT has demonstrated efficacy for anhedonia in the context of depression and anxiety (44), its application to NSSI specifically has not yet been empirically tested. The following proposals should therefore be understood as theoretically motivated directions for future clinical research rather than established treatment protocols.

### 5.3 Restoring the capacity for joy

Beyond the recalibration of reward prediction, effective treatment for the Anhedonic Subtype must address the deficit in savoring. To counter the automatic “dampening” reflex, clinicians may consider employing “Capitalization” techniques. This involves the deliberate sharing of positive events with others to amplify the affective charge. Research suggests that the listener’s “active-constructive responding,” enthusiastic validation of the good news, serves as a social amplifier that extends the duration of dopamine release (45). Furthermore, “Mental Time Travel” protocols allow patients to leverage the brain’s inability to perfectly distinguish between vivid imagination and reality. By guiding patients to vividly visualize past positive memories or anticipate future ones, therapy can artificially stimulate neural reward pathways, strengthening the connectivity of the reward circuit in the absence of external stimuli (46).

Finally, we must address the patient’s immediate need for sensation. Recognizing that the anhedonic patient utilizes NSSI as a form of “forced activation,” we propose that clinicians could explore substituting maladaptive tissue damage with adaptive, high-intensity sensory stimuli. Although sensory-based interventions (e.g., ice immersion) are already used in DBT crisis survival skills, their reframing as “activation” rather than “calming” tools for the Anhedonic Subtype represents a novel clinical hypothesis that warrants controlled investigation. This approach, which we term “Sensory Substitution,” utilizes techniques such as the “mammalian dive reflex” (submerging the face in ice water) or high-intensity interval sprints not to soothe distress, but to generate a physiological jolt. Crucially, the framing of this intervention distinguishes it from standard distress tolerance. The clinician frames the ice water not as a tool to “calm down” (as in Type I interventions), but as a tool to “wake up” the nervous system. This validation of the patient’s need for intensity, rather than the pathologizing of it, builds a therapeutic alliance and provides a safe mechanism to break the paralysis of the void.

We emphasize that the clinical strategies outlined in the preceding two subsections represent theoretically grounded proposals derived from adjacent evidence in affective science and positive psychology. Their efficacy for the Anhedonic Subtype of NSSI has not been directly tested, and rigorous clinical trials will be necessary before these approaches can be recommended for routine practice.

## 6 Conclusion

Nonsuicidal self-injury is not a monolith. While the dominant emotion regulation paradigm correctly identifies “distress relief” as a primary driver for a large cohort of patients, it has inadvertently obscured the silent agony of the Anhedonic Subtype. For these adolescents, self-injury is not a cry of pain, but a “cry for feeling”—a biological imperative to force a blunted reward system into activation via the neurochemical cascade of pain offset. The field must expand its diagnostic lens to accommodate this heterogeneity. Future research should routinely incorporate assessments of anhedonia, such as the Snaith-Hamilton Pleasure Scale (SHAPS), into all risk evaluations to differentiate between “Defensive” and “Deficit” numbness.

Ultimately, the goal of suicide prevention and NSSI treatment cannot be limited to the cessation of bleeding. “Safety” is a necessary but insufficient endpoint for an adolescent trapped in the void. By integrating Positive Affect Treatment, savoring training, and biomarker-informed diagnostics, we can move beyond the mere management of symptoms. We may need to not only teach these young people how not to hurt, but also re-teach them how to feel. The absence of pain is not the presence of life; for the anhedonic adolescent, the true metric of recovery may ultimately be the restoration of the capacity for joy.

## Data Availability

The original contributions presented in the study are included in the article/supplementary material. Further inquiries can be directed to the corresponding author.
